# Clinical characteristics of phenotypes of fecal incontinence

**DOI:** 10.1007/s10151-023-02778-2

**Published:** 2023-03-26

**Authors:** M. E. Knol, E. Bastiaannet, M. C. DeRuiter, H. S. Snijders, J. T. M. van der Heyden, C. I. M. Baeten

**Affiliations:** 1 Department of Surgery, Groene Hart Ziekenhuis, Bleulandweg 10, 2803 HH Gouda, The Netherlands; 2 Department of Anatomy and Embryology, Leiden University Medical Center, Leiden, The Netherlands; 3 Epidemiology, Biostatistics and Prevention Institute, University of Zurich, Zurich, Switzerland

**Keywords:** Fecal incontinence, Proctology, Phenotypes, Surgery

## Abstract

**Purpose:**

Fecal incontinence (FI) is common, but its etiology is complex with large knowledge gaps. Several phenotypes of FI are known, but the phenotype is often not decisive in the chosen therapy. In this study we aimed to assess the association of the clinical characteristics of patients with FI and the various phenotypes, in order to establish a targeted clinical treatment decision tree.

**Methods:**

We retrospectively studied the charts of patients with FI, who visited our institute from January 2018 until December 2020. Patients were divided into the following groups: passive fecal loss, urge incontinence, combined fecal incontinence with predominantly passive fecal loss, and combined fecal incontinence with predominantly urge incontinence. We compared the characteristics between the passive and urge incontinence groups, the passive  and combined mainly passive groups, and the urge and combined mainly urge groups.

**Results:**

Patients with passive incintinence were older, more often had a flaccid anus with presence of a mucosal prolapse, and had a lower resting pressure on anorectal manometry. Patients with urge incontinence were younger and more often had a history of birth trauma. The combined groups showed characteristics of both of the main types of FI.

**Conclusion:**

Differentiating into phenotypes of FI can be clinically meaningful. The patient history and clinical judgement of the consulting specialist, rather than the physical characteristics, seem to be decisive in the categorization. Additional diagnostic testing can be helpful in complicated cases, but should not be used routinely.

## Introduction

Fecal incontinence (FI) is defined as the uncontrolled passage of fecal material that has occurred several times in a month over the past 6 months [1, 2]. Symptoms have a major social impact and patients tend to hide their symptoms from family and healthcare providers [3]. Reported prevalence in current literature varies strongly, ranging from 2% to 21%, and increases with age [4]. FI is a multifactorial problem including altered rectal sensibility, dysfunction of the pelvic floor, and damage to the anal sphincter complex [5, 6]. Although FI is common, its etiology remains complex with major knowledge gaps [3].

Similar to urinary incontinence and irritable bowel syndrome (IBS), FI has different clinical phenotypes, including passive FI, urge FI, and combined FI. Patients with passive FI become aware of solid feces in their underwear without having had an urge [1, 6, 7]. In contrast, patients with urge FI are unable to cease the bowel movement despite an active attempt in response to an urge to defecate [1, 6, 7]. The combined FI is a combination of both phenotypes, in which one of the phenotypes is often more prominent. Prior studies showed that this phenotyping of FI could be clinically meaningful, as urge FI seems to be the result of dysfunction of striated muscle (pelvic floor and external anal sphincter) whereas passive FI could be the result of impairment of the internal anal sphincter [8, 9]. Despite the clinical meaning, and contrary to urinary incontinence and IBS, this phenotyping of FI is often not decisive in targeted therapy.

In this large database study, we aimed to assess the association of the clinical characteristics of patients with FI and the various phenotypes.

## Material and methods

### Patients

From January 2018 until December 2020, a total of 676 patients who visited our outpatient clinic and were registered under an administrational code for FI or pelvic floor dysfunction were identified. We retrospectively studied the charts and patients were included in this study if their complaints met the definition of FI. Patients were excluded if they did not have FI, such as soiling or fecal urgency alone, or if they had FI due to surgical intervention such as low anterior resection syndrome. Soiling was defined as losing moisture only and fecal urgency was defined as a sudden desire to defecate but without an episode of incontinence.

### Settings

All patients were analyzed in our specialist defecation center, where they were examined at a one-stop visit. All patients underwent a structured history taking through a standard questionnaire by two of the authors (JTMH, CIMB) and underwent a thorough physical examination, including rectal and vaginal examination. Through rectal inspection, anatomic pathological changes were examined. With rectal examination an assessment of the anal resting and squeezing pressure was made, as well as an assessment of the function of the pelvic floor, especially the puborectalis muscle. Transanal ultrasound (Aloka, Aloka Europe) was used to assess damage to the sphincter complex. Anorectal manometry was used to measure the anal resting pressure, squeezing pressure, and endurance. In addition, the rectal sensibility was assessed by manometry by recording the first sensation, first sensation of urge, sensation of toileting, and maximum tolerable volume. Both procedures were performed with a 12-French 5-lm anorectal manometry balloon catheter (Medical Measurement Systems, Laborie, the Netherlands) with a standardized pull-through technique. Diagnostic examinations were performed by a specialized nurse practitioner.

### Analysis

Patients were categorized by the consultant surgeon or gastroenterologist into one of four categories: passive fecal loss, urge incontinence, combined fecal incontinence with predominantly complaints of passive fecal loss, or combined fecal incontinence with predominantly complaints of urge incontinence. Firstly, we compared the clinical characteristics between the passive incontinence group and the urge incontinence group. Secondly, we compared the clinical characteristics of the passive incontinence group with the combined mainly passive incontinence  group. Thirdly, we compared the clinical characteristics of the urge inconitnence group with the combined mainly urge incontinence group. Outcome measures were descriptive and reported as numbers with percentages in the case of categorical data, and as a median with an interquartile range in the case of numerical data. The nonparametric equality-of-medians test was used to compare medians between the groups; values equal to the median were split equally between groups. For categorical data, the chi-square test was used; when there were fewer than five values in a cell, the Fisher’s exact test was used. *P* values less than 0.05 were considered statistically significant. STATA/SE 12.0 (Texas, USA) was used to perform the analyses.

## Results

**Fig. 1:**
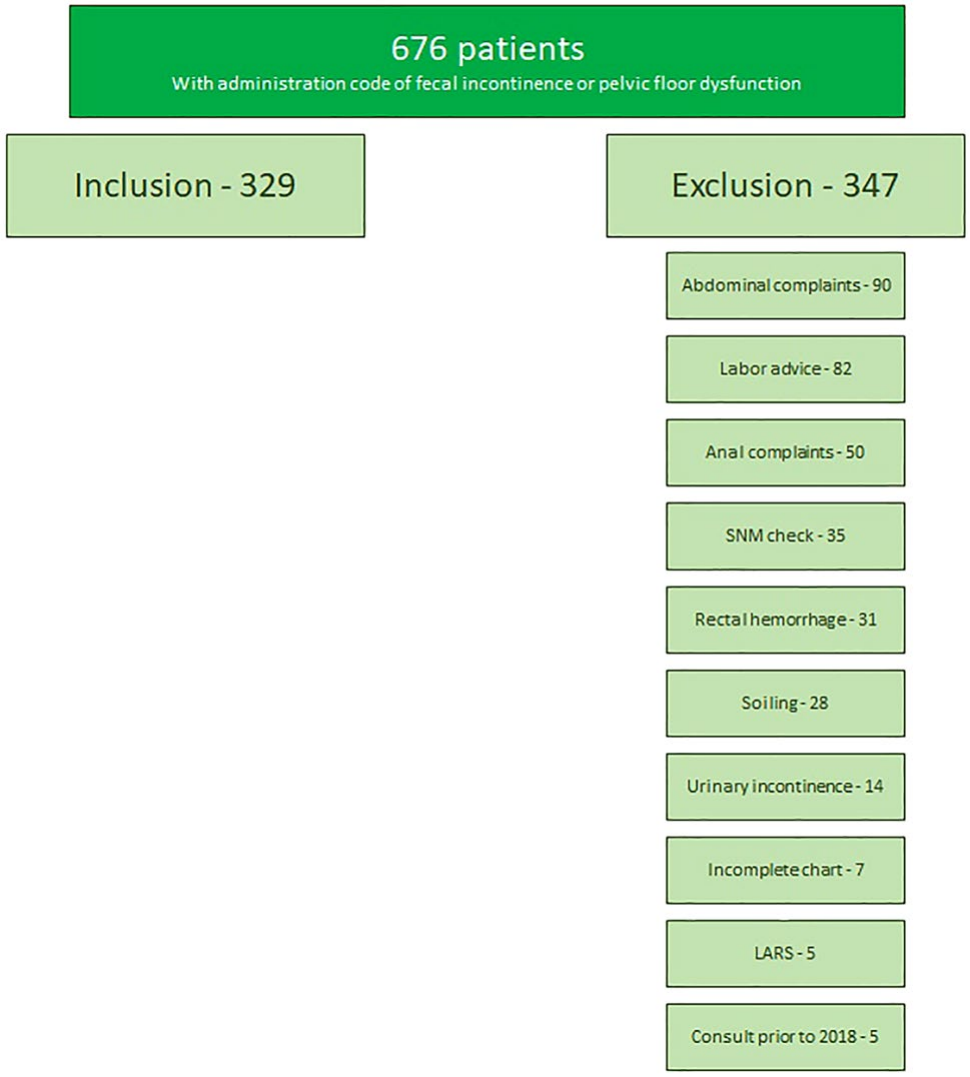
Schedule of inclusion

Of the 676 patients who visited our outpatient clinic between January 2018 and December 2020 with registration of FI or pelvic floor dysfunction, a total of 329 patients met the inclusion criteria; 347 patients were excluded (Fig. 1). Ninety patients were excluded because of abdominal complaints other than FI (e.g., obstipation, stomach ache). Fifty patients had anal complaints (e.g., fissures or hemorrhoids). Eighty-two patients received advice concerning labor following a third- or fourth-degree tear. Thirty-five patients were annually checked following sacral neuromodulation (SNM) implantation elsewhere. Thirty-one patients were excluded because of rectal hemorrhage. Twenty-eight patients had complaints of soiling. Fourteen patients received percutaneous tibial nerve stimulation (PTNS) because of urinary incontinence. Seven patients were excluded because of an incomplete chart. Five patients were excluded because of complaints of low anterior resection syndrome (LARS). The last five patients were excluded because their first consultation was before January 2018. Of the 329 patients who met the inclusion criteria, 98 (30%) patients suffered from only passive incontnence, 126 (38%) patients suffered from only urge incontinence, 62 (19%) patients suffered from combined but mainly urge incontinence, and 43 (13%) patients suffered from combined but mainly passive incontinence.

### Passive incontinence group compared with the urge incontinence group

The median age of patients with pure PFL was significantly higher than for patients with pure urge incontinence (70 vs 64 years, *P* = 0.005, Table 1). There were no significant differences in gender, diabetes, and prior abdominal, anal, or gynecological surgery between the two groups. Women with only urge incontinence more often had a vaginal tear in their medical history (*P* = 0.03). They also reported issues for more than 5 years more frequently than  patients with only passive incontinence (53% vs 37%, *P* = 0.02). In the passive incontinence group, most patients reported only loss of faecal fluid, whereas in the group with urge incontinence most patients reported solid faecal leakage. Overall, 68% of the patients with passive incontinence reported no urgency compared to 3% of the patients with urge incotinence (*P* < 0.001). Patients with passive incontnence more frequently had a flaccid anus (29% vs 14%, *P* = 0.012) and the presence of a mucosal prolapse (59% vs 24%, *P* < 0.001). A flaccid anus was defined as a lax perineal body that could be retracted almost to the pubic. Physical examination revealed no difference in anal resting or squeezing pressure. In addition, patients with passive incontinence more often had a normally functioning puborectal muscle (71% vs 51%, *P* = 0.015). Transanal ultrasound detected no differences in internal or external anal sphincter defects. Anorectal manometry showed a significantly higher median anal resting pressure in patients with urge incontinence (40 mmHg vs 55 mmHg, *P* = 0.001). No significant difference was seen in anal squeezing pressure, but both groups had a lower than normal median squeezing pressure (30 mmHg vs 25 mmHg). In addition, no significant difference was seen in rectal sensibility.

**Table 1:** Passive fecal loss (PFL) versus urge incontinence (UI)

	Passive faecal leakage (98)	Urge incontinence (126)	*P*
Age, median (IQR)	70 (62–78)	64 (53–72)	0.005*
Gender, *n* (%)			
Man	15 (15.3)	28 (22.2)	0.19
Woman	83 (84.7)	98 (77.8)	
Diabetes, *n* (%)			
No	90 (92.8)	110 (87.3)	0.18
Yes	7 (7.2)	16 (12.7)	
Abdominal/anal surgery, *n* (%)			
No	57 (58.8)	80 (63.5)	0.47
Yes	40 (41.2)	46 (36.5)	
Gynecological surgery, *n* (%)			
No	32 (33.0)	40 (32.0)	0.41
Yes	50 (51.5)	57 (45.6)	
Vaginal tear, *n* (%)			
No	41 (46.1)	36 (30.0)	0.03*
Yes	26 (29.2)	54 (45.0)	
Complaints, *n* (%)			
< 3 months	1 (1.1)	2 (1.6)	0.02*
3 months–1 year	25 (28.1)	15 (12.4)	
1–5 years	30 (33.7)	40 (33.1)	
> 5 years	33 (37.1)	64 (52.9)	
Amount of fecal loss, *n* (%)			
Unclear	19 (21.6)	22 (20.9)	< 0.001*
Moisture only	37 (42.0)	17 (16.2)	
Smaller part of portion	17 (19.3)	19 (18.1)	
Reasonable amount of portion	13 (14.8)	26 (24.8)	
Entire portion	2 (2.3)	21 (20.0)	
Flaccid anus, *n* (%)			
No	67 (71.3)	104 (85.3)	0.012*
Yes	27 (28.7)	18 (14.7)	
Mucosal prolapse, *n* (%)			
No	39 (41.0)	93 (76.2)	< 0.001*
Yes	56 (59)	29 (23.8)	
Physical examination: anal resting pressure, *n* (%)			
High	3 (3.4)	8 (6.7)	0.51
Normal	43 (48.3)	59 (49.6)	
Low	43 (48.3)	52 (43.7)	
Physical examination: anal squeezing pressure			
High/normal	30 (34.9)	36 (31.3)	0.80
Low	56 (65.1)	79 (68.7)	
Physical examination: puborectal muscle function			
Normal	62 (70.5)	60 (50.9)	0.015*
Bad	17 (19.3)	47 (39.8)	
Paradoxal	9 (10.2)	11 (9.3)	
Additional examination: anal resting pressure, mmHg (IQR)	40 (30–50)	55 (50–70)	< 0.001*
Additional examination: anal squeezing pressure, mmHg (IQR)	30 (18–42)	25 (13–41)	0.19
Additional examination: rectal sensibility, *n* (%)			
Hyper sensible	5 (10.6)	30 (28.0)	0.058
Normal	15 (31.9)	26 (24.3)	
Hypo sensible	27 (57.5)	51 (47.7)	

*Statistically significant, i.e., *P* < 0.05. **Due to the retrospective study design, not all parameters were available per patient and some data was missing.

### Passive incontinence group compared with the combined group with mainly passive incontinence

There were no significant differences in age, gender, diabetes, and prior abdominal, anal, or gynecological surgery between the two groups. Patients with combined incontinence more often reported labor involving vacuum extraction or forceps (*P* = 0.04, Table 2). A larger percentage reported urgency before the fecal loss in the combined group (31.8% vs 75.6%, *P* < 0.001). Physical examination more often showed a flaccid anus in the combined group (29% vs 51%, *P* = 0.011), a lower anal resting pressure (48% vs 78%, *P* = 0.005), a lower squeezing pressure (65% vs 92%, *P* = 0.006), and a less effective puborectal muscle (30% vs 51.3%, *P* = 0.005). No differences were shown in anorectal ultrasound or anal resting pressure with anorectal manometry. The median anal squeezing pressure was significantly lower in the combined group (30 mmHg [18–42] vs 20 mmHg [10–35], *P* = 0.046]. Rectal sensibility was not significantly different between groups.

**Table 2:** Passive fecal loss (PFL) versus combined fecal incontinence with predominantly passive fecal loss

	Passive faecal incontinence only (98)	Combined incontinence with mainly passive leakage (43)	*P*
Age, median (IQR)	70 (62–78)	71 (56–77)	0.81
Gender, *n* (%)			
Man	15 (15.3)	2 (4.6)	0.07
Woman	83 (84.7)	41 (95.4)	
Diabetes, *n* (%)			
No	90 (92.8)	37 (86.1)	0.20
Yes	7 (7.2)	6 (13.9)	
Abdominal/anal surgery, *n* (%)			
No	57 (58.8)	27 (62.8)	0.65
Yes	40 (41.2)	16 (37.2)	
Gynecological surgery, *n* (%)			
No	32 (33.0)	14 (32.5)	0.35
Yes	50 (51.5)	26 (60.5)	
Vacuum extraction/forceps delivery, *n* (%)			
No	59 (64.8)	26 (61.9)	0.04*
Yes	10 (11.0)	11 (26.2)	
Urge prior to defecation, *n* (%)			
Yes, but too late	15 (17.0)	20 (48.8)	< 0.001*
Yes, but mostly too late	13 (14.8)	11 (26.8)	
No	60 (68.2)	10 (24.4)	
Flaccid anus, *n* (%)			
No	67 (71.3)	21 (48.8)	0.011*
Yes	27 (28.7)	22 (51.2)	
Mucosal prolapse, *n* (%)			
No	39 (41.0)	24 (55.8)	0.11
Yes	56 (59)	19 (44.2)	
Physical examination: anal resting pressure, *n* (%)			
High	3 (3.4)	0 (0.0)	0.005*
Normal	43 (48.3)	9 (21.9)	
Low	43 (48.3)	32 (78.1)	
Physical examination: anal squeezing pressure			
High/normal	30 (34.9)	3 (7.9)	0.006*
Low	56 (65.1)	35 (92.1)	
Physical examination: puborectal muscle function			
Normal	62 (70.5)	19 (48.7)	0.005*
Bad	17 (19.3)	19 (48.7)	
Paradoxal	9 (10.2)	1 (2.6)	
Additional examination: anal resting pressure, mmHg (IQR)	40 (30–50)	38 (25–55)	0.82
Additional examination: anal squeezing pressure, mmHg (IQR)	30 (18–42)	20 (10–35)	0.046*
Additional examination: rectal sensibility, *n* (%)			
Hyper sensible	5 (10.6)	12 (31.6)	0.06
Normal	15 (31.9)	9 (23.7)	
Hypo sensible	27 (57.5)	17 (44.7)	

*Statistically significant, i.e., *P* < 0.05. **Due to the retrospective study design, not all parameters were available per patient and some data was missing

### Urge incontinence compared with the group with combined but mainly urger incontinence

There were no significant differences in age, diabetes, and prior abdominal or anal surgery between the two groups. The urge incontinence group contained significantly more men (22% vs 8%, *P* = 0.02, Table 3) and had significantly fewer prior gynecologic surgeries in the past (46% vs 61%, *P* = 0.02). With physical examination, the combined group more often had a flaccid anus (15% vs 46%, *P* < 0.001), more often had the presence of a mucosal prolapse (24% vs 47%, *P* = 0.002), more often had a lower anal resting pressure (44% vs 72%, *P* < 0.001), and more often had a lower squeezing pressure (69 vs 83%, *P* = 0.026). No difference was seen in the function of the puborectal muscle. Transanal ultrasound showed no difference in the presence of an anal sphincter defect. Anorectal manometry showed a significantly lower median anal resting pressure in the combined group (55 mmHg [40–70] vs 40 mmHg [30–55], *P* = 0.001). No difference was seen in anal squeezing pressure and rectal sensibility with anorectal manometry.

**Table 3:** Urge incontinence versus combined fecal incontinence with predominantly urge incontinence

	Urge incontinence (126)	Combined mainly urge incontinence (62)	*P*
Age, median (IQR)	64 (53–72)	67.5 (60–72)	0.16
Gender, *n* (%)			
Man	28 (22.2)	5 (8.1)	0.02*
Woman	98 (77.8)	57 (91.9)	
Diabetes, *n* (%)			
No	110 (87.3)	50 (80.7)	0.23
Yes	16 (12.7)	12 (19.3)	
Abdominal/anal surgery, *n* (%)			
No	80 (63.5)	34 (54.8)	0.25
Yes	46 (36.5)	28 (45.2)	
Gynecological surgery, *n* (%)			
No	40 (32.0)	20 (32.3)	0.02*
Yes	57 (45.6)	38 (61.3)	
Flaccid anus, *n* (%)			
No	104 (85.3)	33 (54.1)	< 0.001*
Yes	18 (14.7)	28 (45.9)	
Mucosal prolapse, *n* (%)			
No	93 (76.2)	32 (53.3)	0.002*
Yes	29 (23.8)	28 (46.7)	
Physical examination: anal resting pressure, *n* (%)			
High	8 (6.7)	0 (0.0)	0.001*
Normal	59 (49.6)	17 (28.3)	
Low	52 (43.7)	43 (71.7)	
Physical examination: anal squeezing pressure			
High/normal	36 (31.3)	10 (17.2)	0.026*
Low	79 (68.7)	48 (82.8)	
Physical examination: puborectal muscle function			
Normal	60 (50.9)	32 (55.2)	0.89
Bad	47 (39.8)	22 (38.0)	
Paradoxal	11 (9.3)	4 (6.9)	
Additional examination: anal resting pressure, mmHg (IQR)	55 (50–70)	40 (30–55)	0.001*
Additional examination: anal squeezing pressure, mmHg (IQR)	25 (13–41)	22.5 (10–40)	0.70
Additional examination: rectal sensibility, *n* (%)			
Hyper sensible	30 (28.0)	15 (25.9)	0.77
Normal	26 (24.3)	12 (20.7)	
Hypo sensible	51 (47.7)	31 (53.4)	

*Statistically significant, i.e., *P* < 0.05. **Due to the retrospective study design, not all parameters were available per patient and some data was missing

## Discussion

In this study, patients with passive FI were older, more often had a flaccid anus with presence of a mucosal prolapse, and had a lower resting pressure on anorectal manometry. Patients with urge incontinence were younger and more often had a history of birth trauma. No other physical characteristics were linked to this subtype. According to their medical history, the urge incontinence group reported longer existing complaints and quantitatively more loss. Our analysis confirmed that the combined groups show characteristics of both of the two main types. This suggests there are distinguishable phenotypes with varying symptomatology and diagnostic results. These findings aid further treatment.

Although rare, some articles about the clinical characteristics of FI have been published. Gee and Durdey concluded that urge incontinence indicates severe external anal sphincter dysfunction, and incontinence without urge is associated with impairment of the internal anal sphincter and reduction in resting anal pressure [9]. Pahwa et al. reported weaker squeezing pressure on digital examination, hypersensitivity on anorectal manometry, and a trend toward the presence of an external anal sphincter defect in patients with urge incontinence. Women with passive FI had lower resting tone on digital rectal examination, significantly lower mean anal resting pressures on anorectal manometry, and a trend towards  more frequent presence of an internal anal sphincter defect [8]. Our results concerning passive FI are largely consistent with these previous studies. However, we did not report any significant differences in the presence of an internal anal sphincter defect. The results concerning urge incontnence were more inconsistent with the previous literature. We did not identify any difference in external anal sphincter defect frequency. We did observe a low squeezing pressure, but this did not differ significantly from the patients with passive incontinence only.

We did observe, that the patients with urge incontinence more often had a vaginal tear in the past and the patients with combined  but mainly passive incontinence more often had a forceps or vacuum-assisted delivery. The fact that these patients more often had a birth trauma without physical damage to the anal sphincters could imply that the damage after delivery is more neurological in nature. This could be an explanation why SNM is equally effective in patients with FI with and without a sphincter defect and after sphincter repair [10, 11].

The lack of distinctive characteristics in additional diagnostics could be due to the wide range of what is considered normal. This is particularly the case with anorectal manometry, and measurements are influenced by gender and age [12]. This might explain why we could not find any significant differences except the lower median anal resting pressure in patients with passive FI. In any case, the current literature addresses mainly resting pressure and squeezing pressure. Future research should perhaps focus on combination of sphicnter pressures  e.g. can a high squeezing pressure compensate for a low resting pressure or vice versa? What would be an ideal combination and should we consider the closing pressure (resting pressure plus squeezing pressure) as an additional measurement?

Our study has a two significant strengths. It has a large sample size and we have distributed patients into four groups instead of the usual classification into passive and urge incontinence. A limitation is the retrospective study design. In this study, we used a standardized questionnaire. However, we used the judgement of the consultant surgeon or gastroenterologist to categorize the patients into one of the four groups. This explains why a number of patients stated they only lost some moisture, more consistent with soiling, but were categorized into one of the four groups on the basis of additional questions of the consultant gastroenterologist or surgeon. One can conclude that the questionnaire cannot replace a specialist assessment.

Altogether, our findings suggest that differentiating into types of FI can be clinically meaningful. Particularly, the patient history and clinical judgement of the consulting specialist, rather than the physical characteristics, seem to be decisive in the categorization. Additional diagnostic testing could be helpful in more complicated cases, but essential for a diagnosis. Our results are a route towards a more pattern specific approach to the management of FI.

## Data Availability

Not applicable.
